# Huge thrombus in the ascending aorta: a case report and literature review

**DOI:** 10.1186/s13019-019-0975-y

**Published:** 2019-09-04

**Authors:** Baogang Wang, Dashi Ma, Dianbo Cao, Xiaxia Man

**Affiliations:** First Hospital Jilin University, Changchun, 130021 People’s Republic of China

**Keywords:** Floating thrombus, Ascending aorta, Transthoracic echocardiography, Computed tomography angiography

## Abstract

**Background:**

A floating thrombus in the ascending aorta is occasionally found in clinical practice. The treatment for such lesions is poorly defined and mainly depends on the clinical experience of the surgeons.

**Case presentation:**

We herein report a case involving a 22- × 22- × 45-mm space-occupying lesion in the ascending aorta. The patient was successfully treated with surgical intervention. Thrombectomy and ascending aorta replacement were performed to prevent systemic embolization. Histopathological examination revealed that the lesion was a thrombus.

**Conclusions:**

Aortic computed tomography angiography is a useful examination technique for patients with aortic thrombi. Resection of the thrombus can effectively reduce the risk of recurrent embolism.

## Background

A floating thrombus in the ascending aorta is occasionally found in clinical practice. The treatment for such lesions is poorly defined and mainly depends on the clinical experience of the surgeons. Considering the catastrophic effects of embolism, emergency surgery is usually needed. The ethics committee of the Jilin University First Hospital approved this study and the publication of this case report. Informed consent was obtained from the patient.

## Case presentation

**Fig. 1:**
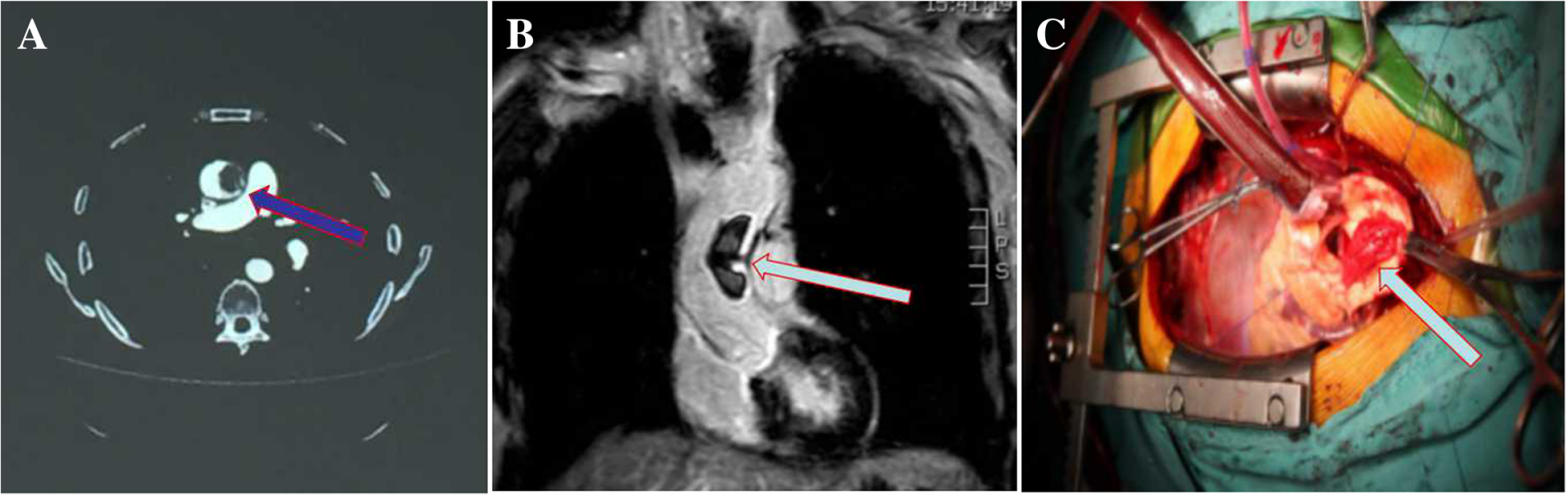
**a**: Computed tomography showed a large lesion atteched to the aortic inner wall. **b**: Magnetic resonance investigation showed that the feature of the lesion might be thrombus. **c**: Intraoperative photography after excision showed the in situ mass attached to the aortic wall (the blue arrow)

A 56-year-old, 61-kg, 168-cm man was admitted to our institution for a routine physical examination. His vital signs were as follows: pulse, 100 beats/min, blood pressure, 140/100 mmHg; oxygen saturation, 95% on room air; and body temperature, 36.5 °C. No murmurs, dyskinesia or paresthesia were detected. The patient had no history of atrial fibrillation. His serum N-terminal pro-brain natriuretic peptide concentration was slightly elevated (131 pg/ml), and his prothrombin time was 10 s. No abnormalities were found in the complete blood count or tumor markers. The serum procalcitonin level was 0.40 ng/ml, the cardiac troponin I level was 0.012 ng/ml, the erythrocyte sedimentation rate was 12 mm/h, and the C-reactive protein level was 3.10 mg/L. A blood culture was negative. Transthoracic echocardiography (TTE) and computed tomography angiography (CTA) revealed a well-defined pedunculated mass measuring 22 × 22 × 45 mm in ascending aortic lumen (Fig. 1a). Magnetic resonance imaging showed multiple asymptomatic cerebral infractions. No abnormalities were found on color Doppler ultrasound of the lower extremities. Furthermore, magnetic resonance imaging demonstrated atherosclerosis of the ascending aorta, and the mass was suspected to be thrombus (Fig. 1b).

**Fig. 2:**
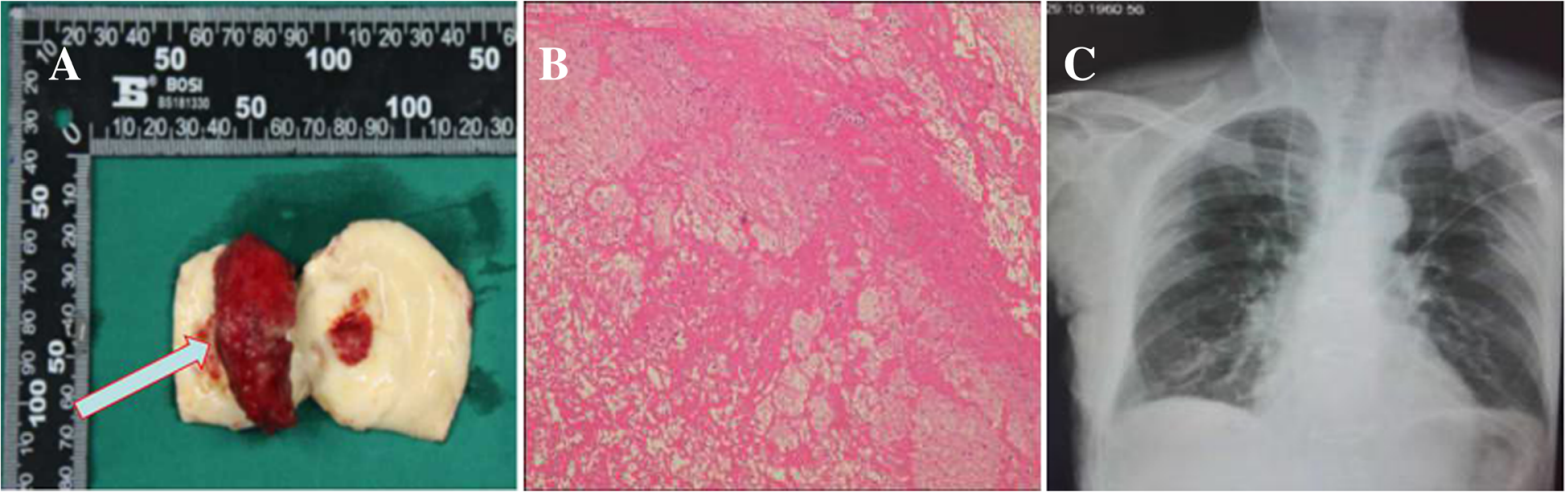
**a**: Intraoperative photography after excision showed mass specimen was well defined (the blue arrow). **b**: Hematoxylin-eosin stained specimen showed the mass was thrombus (40×). **c**: Postoperation X-ray showed that the outline of the heart and the aorta were normal

The patient underwent a thrombectomy. Cardiopulmonary bypass was established by cannulation of the right femoral artery and right atrium. The ascending aorta was clamped proximal to the brachiocephalic trunk. A thrombus that was attached to the aortic wall by a stalk was located approximately 3 cm above the aortic annulus (Figs. 1c, 2a). The thrombus was removed. The implant site appeared to be an atherosclerotic plaque with extensive ulceration. The aortic valve was preserved. There was no sign of endocarditis, aortic valve insufficiency, or other pathological findings. The ascending aorta was replaced with a 24-mm-diameter artificial vessel (Woven Double Velour Vascular Graft; Maquet Cardiovascular LLC, Wayne, NJ, USA).

Histopathological examination revealed that the mass was a thrombus (Fig. 2b). The postoperative course was uneventful. No recrudescence or concomitant visceral or vascular embolism was observed. The patient was discharged 11 days after surgery. The patient was treated with oral aspirin for 6 months postoperatively.

## Discussion

Although a thrombus in the aorta is not the major source of emboli in patients with ischemic stroke, the symptoms of aortic thrombi are usually associated with embolism caused by shedding of the thrombus. These symptoms include unconsciousness caused by ischemic stroke, abdominal pain caused by splenic infarction, and lower limb pain caused by acute ischemia [1]. Surgeons should consider widening the investigation of the primary lesion. Surgical resection of the primary lesion should be performed as soon as possible. The diagnosis of a thrombus in the aorta depends on imaging studies including CTA, echocardiography, and magnetic resonance imaging. No treatment guidelines for an aortic thrombus are currently available. If the lesion is identified as a thrombus before the operation and is located in the descending aorta, abdominal aorta, or its branches, most surgeons prefer to use anticoagulants [2, 3] and implant an endovascular stent in the descending aorta [4]. Anticoagulants include low-molecular-weight heparin, apixaban, and warfarin. In the present case, repeat TTE and CTA examinations demonstrated a reduction in the size of the thrombus. The international normalized ratio was simultaneously monitored. If a floating lesion demonstrated by TTE and aortic CTA [5, 6] is located in the ascending aorta, most surgeons prefer to perform thrombectomy on cardiopulmonary bypass. The postoperative course involves treatment of complications caused by the embolism. A notable feature of the present case is that the patient exhibited no preoperative symptoms caused by the embolism. CTA examination of the thoracoabdominal aorta also showed no asymptomatic renal or splenic infarction. A 3.0-cm distance was present between the distal end of the clot and the origin of the brachiocephalic artery trunk. The clot did not extend into the arch. Considering the risk of concomitant visceral or vascular embolism in this case, we performed an aortotomy on cardiopulmonary bypass [7]. The cardioplegia was administered directly to the coronary arteries after the aortotomy. The aortic cross-clamp time was 50 min, and the level of hypothermia was 34.0 °C. No embolism-related complications occurred postoperatively.

Aortic CTA is recommended as the first-choice examination because of its convenience and high sensitivity. The prognosis of an aortic thrombus is poor, but it may be improved by prompt diagnosis and treatment using aortic CTA [1]. This case highlights the importance of a thorough investigation, especially for asymptomatic patients. Preoperative aortic CTA can accurately reveal the location and shape of space-occupying lesions for surgeons [8]. Aortic CTA may simultaneously indicate the presence of other aortic diseases, such as aortic aneurysm and aortic dissection. This will determine the final surgical procedures and the extent of resection. When the patient has a tortuous femoral artery, surgeons prefer to perform cannulation at the axillary artery. The imaging offered by aortic CTA is helpful in choosing the technique for establishing cardiopulmonary bypass. Aortic CTA plays an important role in every aspect of the diagnosis and treatment of an aortic thrombus.

The etiology of a thrombus in the aorta is complex. Aortic thrombi can be caused by blood disorders (e.g., protein S or protein C deficiency and anti-phospholipid antibody syndrome), tumors, aortitis, collagen disease, aortic structural abnormalities (e.g., aortic aneurysms), intra-aortic atheroma, hormone therapy, steroid use, and atrial fibrillation [9]. The present patient had no evidence of coagulation disorders or arteriosclerotic risk factors such as hypertension or diabetes mellitus. Thus, this case indicates that thrombosis may develop in patients without traditional risk factors. Based on the surgical outcome in the present case, we speculate that the thrombus had been caused by recurrent bleeding after rupture of the nutrient artery of the aortic intima. In most cases, the hematoma breaks through the inner wall of the aorta and becomes an acute aortic dissection. Some patients are treated with medication for several weeks, and the hematoma is gradually absorbed. According to a study based on computational fluid dynamics carried out by Zhang [10], the distributions of the hemodynamic variables during the cardiac cycle showed spatiotemporal differences. The flow velocity and shear stress gradually increased from the ascending aorta to the origin of the left subclavian artery and from the outer to inner wall of the aortic artery, and these parameters were under the influence of anatomical factors such as vascular branching. The shear stress was higher at the site of vascular branching. We speculate that the hematoma was restricted by the surrounding tissues in our patient, and no aortic dissection developed. The volume of the hematoma gradually increased after repeated hemorrhage, and a thrombus formed in the ascending aorta. The shed thrombus led to cerebral infarction.

## Conclusions

A floating thrombus in the ascending aorta may develop in patients without traditional risk factors. Aortic CTA is a useful examination technique for patients with aortic thrombi. Thrombectomy can effectively reduce the risk of recurrent embolism.

## Data Availability

Not applicable.

## Abbreviations

CTA: computed tomography angiography
TTE: transthoracic echocardiography
